# Diagnostic Value of Serum Golgi Protein 73 for Liver Inflammation in Patients with Autoimmune Hepatitis and Primary Biliary Cholangitis

**DOI:** 10.1155/2022/4253566

**Published:** 2022-01-15

**Authors:** Mingjie Yao, Leijie Wang, Jianwen Wang, Yanna Liu, Shuhong Liu, Jingmin Zhao, Fengmin Lu

**Affiliations:** ^1^Department of Anatomy and Embryology, School of Basic Medical Sciences, Peking University Health Science Center, Beijing 100191, China; ^2^Department of Microbiology & Infectious Disease Center, School of Basic Medicine, Peking University Health Science Center, 38 Xueyuan Road, Beijing 100191, China; ^3^Department of Pathology and Hepatology, The Fifth Medical Center of PLA General Hospital, 100039, China; ^4^Center of Precision Medicine, Academy of Medical Sciences, Zhengzhou University, Zhengzhou, China; ^5^Peking University People's Hospital, Peking University Hepatology Institute, Peking University Health Science Center, 11 South Xizhimen Street, Beijing 100044, China

## Abstract

**Background:**

There is lack of reliable serum biomarkers to reflect the severity of liver necroinflammation for those who suffer autoimmune liver diseases (AILDs). In this study, a previously established patient cohort was used to explore the potential of serum Golgi protein 73 (GP73) as a noninvasive marker of AILD-related liver necroinflammation.

**Methods:**

Serum GP73 concentration was measured in a retrospective cohort of 168 AILD patients, which included 74 patients with autoimmune hepatitis (AIH) and 94 with primary biliary cholangitis (PBC) who had undergone liver biopsy. Spearman's correlation and multivariate analysis were used to evaluate the relationship between serum GP73 and liver necroinflammation. A receiver operating characteristic curve was constructed to evaluate the value of GP73 for the prediction of moderate or severe liver necroinflammation. The diagnostic value of serum GP73 was also compared with that of alkaline phosphatase (ALP) in patients with PBC. Histologically, immunohistochemical analysis was performed to assess hepatic GP73 expression.

**Results:**

Both the serum level and hepatic tissue expression of GP73 protein were aberrantly elevated and correlated well with the severity of necroinflammation in both AIH (rho = 0.655, *P* < 0.001) and PBC (rho = 0.547, *P* < 0.001) patients. The results here suggested that serum GP73 could be an independent biomarker to reflect the severity of liver necroinflammation. The AUROCs for GP73 to predict moderate necroinflammation (≥G2) and severe necroinflammation (≥G3) in patients with AIH were 0.828 and 0.832, respectively. Moreover, the AUROCs of serum GP73 for the identification of moderate necroinflammation (≥G2) (AUROC = 0.820, *P* < 0.001) and severe necroinflammation (≥G3) (AUROC = 0.803, *P* < 0.001) were superior to those of ALP (≥G2: AUROC = 0.607, *P* = 0.028 and ≥G3: AUROC = 0.559, *P* = 0.357) in patients with PBC. Mechanically, interlukin-6 (IL-6), the proinflammatory and prohepatic regenerating cytokine, could transcriptionally upregulate GP73 gene expression.

**Conclusion:**

Serum GP73 is a potential noninvasive biomarker to evaluate the severity of liver necroinflammation in patients with AILDs.

## 1. Introduction

Autoimmune hepatitis (AIH) and primary biliary cholangitis (PBC) are autoimmune-mediated inflammatory liver diseases that involve different hepatic parenchymal cells: primarily hepatocytes in AIH and biliary epithelial cells in PBC. Each can progress to liver fibrosis, cirrhosis, and liver failure. Liver necroinflammation is closely related to the prognosis of AIH and PBC. Serum alanine aminotransferase (ALT) and alkaline phosphatase (ALP) activities specifically reflect liver necroinflammation which is closely related to prognosis [1, 2]. Therefore, the treatment of these diseases is aimed at to reducing ALT and ALP activities, slowing disease progression, and improving clinical outcomes. However, these biomarkers primarily reflect disease activity but not disease progression. Monitoring liver necroinflammation and scoring fibrosis stage would be very helpful for therapeutic decision-making and treatment efficacy evaluation, which is usually required over a long period of time. Although liver biopsy is the gold-standard method for the diagnosis of AILDs, it has notable disadvantages and cannot be conducted time after time [3]. Finding new reliable noninvasive biomarkers is an unmet clinical need for AIH and PBC management.

Golgi protein 73 (GP73) is a resident membrane protein of unknown function that is expressed in biliary epithelial cells but is absent or expressed at very low levels in normal hepatocytes and might be involved in inflammatory liver diseases [4–11]. Previous studies have demonstrated the potential utility of serum GP73 concentration in the assessment of the disease progression in chronic hepatitis B [4, 5, 8, 12, 13], [14], and our own previous findings have demonstrated that serum GP73 represents a surrogate biomarker of liver fibrosis, regardless of the disease etiology [15]. We reported that serum GP73 is closely related to the severity of liver necroinflammation and has the potential to identify moderate to severe liver necroinflammation in patients with either chronic hepatitis B virus infection or nonalcoholic steatohepatitis (NASH) [16–18]. In this study, we aimed to explore the relationship between GP73 and liver necroinflammation in AILDs, and we evaluated the potential value of serum GP73 as a noninvasive biomarker of liver inflammation in patients with AIH or PBC. Moreover, using lipopolysaccharide- (LPS-) induced liver injured C57/BL6J mouse model, the molecular mechanism relevant to aberrant elevation of serum GP73 and its association with liver inflammation was explored.

## 2. Materials and Methods

### 2.1. Patients

Between January 2011 and March 2019, 43 patients from Beijing 302 Hospital and 125 patients from Shanghai Renji Hospital with confirmed AILDs were recruited, including 74 patients with AIH and 94 patients with PBC; the diagnosis was made based on the established criteria for AIH [19] and PBC [20], respectively. In addition, the serum GP73 concentration was measured in 121 healthy volunteers who had a body mass index (BMI) ≤ 28 kg.m^2^, did not have any acute or chronic diseases, and did not have hyperlipidemia, diabetes, or hypertension.

### 2.2. Clinical Indices

The clinical data including the serum total bilirubin (TBIL), immunoglobulin G (IgG), and immunoglobulin M (IgM) of each enrolled patient were collected, and serum GP73 concentration was measured using a Golgi protein 73 assay kit (Hotgen Biotech Inc., Beijing, China).

### 2.3. Liver Biopsy and Histology

Liver biopsies were stained with hematoxylin and eosin (HE) and Masson's trichrome. The grade of liver fibrosis was scored at 0–4, according to the METAVIR scoring system [21, 22]. The grade of liver necroinflammation was scored at 0–4, according to Scheuer's scoring system.

### 2.4. GP73 Immunohistochemistry

Immunohistochemical staining was performed as described previously using an anti-GP73 antibody (ab109628, 1 : 1,000 dilution; Abcam, Cambridge, UK).

### 2.5. Mouse Model and Real-Time PCR

To mimic acute hepatic injury, C57/BL6J mice were intraperitoneally injected with 10 mg/kg LPS. Mice were sacrificed after injection at 6 hours, 12 hours, 24 hours, 48 hours, and 72 hours. For the blocking experiment, 2 *μ*g/g anti-mouse IL-6 antibody (504506, BioLegend, USA) was intravenously injected into the tail simultaneously with LPS injection, while the control group was intravenously injected with 2 *μ*g/g rat IgG1 (400432, BioLegend, USA). For the inhibition experiment, mice were intragastrically filled with 30 mg/kg ruxolitinib (S1378, SelleckChem, USA) simultaneously with LPS injection, while the control group was intragastrically filled with PBS-diluted DMSO. Mice were sacrificed at 6 hours and 12 hours after injection. Part of the liver tissue was stored in a -80°C freezer for RNA extraction. Liver tissue was ground in liquid nitrogen. Then, RNA was extracted with the TRIzol reagent (15596018, Invitrogen, USA). Reverse transcription was performed by using the Transcriptor cDNA Synth. Kit (4897030001, Roche, USA). With GAPDH as the housekeeping gene, the mRNA levels of IL-6 and GP73 were quantitatively measured by RT-PCR using the FS Universal SYBR Green Master Mix (4913914001, Roche, USA). The primers are listed in Supplementary Table 1.

### 2.6. Statistical Analysis

Data were analyzed using IBM SPSS v.22.0 software (International Business Machines Corporation, Armonk, NY, USA) and GraphPad Prism version 5.0 (GraphPad Software Inc., La Jolla, CA, USA). The differences between the groups were evaluated using ANOVA, following rank transformation or *t*-tests, according to the distribution of data. Spearman's rank correlation was used to describe the relationship between two variables. Multivariate analysis using a stepwise method, with a *P* value for slentry of 0.2 and *P* value for slstay of 0.05, was performed to select and eliminate variables. The diagnostic accuracies of GP73 and ALP were compared by calculating the area under the receiver operating characteristic (AUROC) curve and a 95% confidence interval (CI) using MedCalc (15.6.1). All tests of significance were two-tailed, and *P* < 0.05 was considered to represent statistical significance.

## 3. Results

### 3.1. Patient Characteristics

In this retrospective study, a total of 168 AIH or PBC patients diagnosed between January 2011 and March 2019, who underwent liver biopsy and met the study criteria, were enrolled. A complete set of clinical data, including ALP, was available for 84/94 patients. The patients' clinical characteristics are shown in Table 1.

### 3.2. Serum and Hepatic GP73 Levels Are Significantly Higher in Patients with AILD-Related Liver Necroinflammation

**Figure 1:**
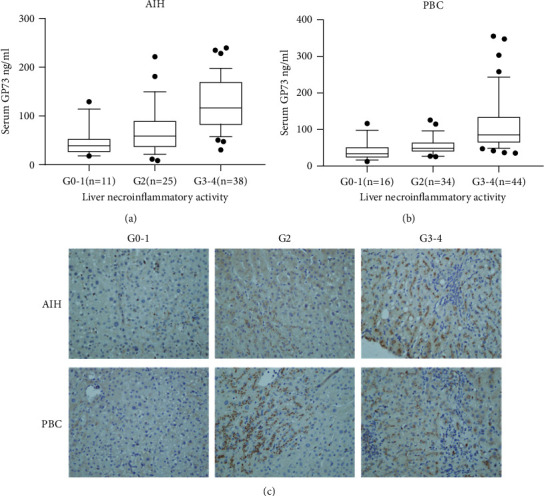
Serum GP73 (a, b) and hepatic GP73 (c) levels increase with the severity of liver necroinflammation.

To investigate whether serum GP73 has potential value for the prediction of liver necroinflammation in patients with AIH or PBC, the serum GP73 levels in patients with AIH or PBC were measured. The results showed a gradual but distinguishing increase in serum GP73 concentration in parallel with the increase in severity of liver necroinflammatory damage (Figures 1(a) and 1(b)), most obviously in patients with AIH (*P* < 0.001) and to some extent in patients with PBC (*P* < 0.001). In detail, the median serum GP73 concentrations in AIH patients at different grades were 39.0 ng/mL (G0–1), 58.8 ng/mL (G2), and 116.5 ng/mL (G3–4), which indicated a positive correlation of serum GP73 levels with the severity of liver necroinflammation. A similar tendency was observed in patients with PBC, in whom the median serum GP73 concentrations were 33.9 ng/mL (G0–1), 50.5 ng/mL (G2), and 85.6 ng/mL (G3–4). In line with these findings, hepatic GP73 expression also increased with the severity of necroinflammation in patients with AIH or PBC (Figure 1(c)).

### 3.3. Serum GP73 Is an Independent Predictor of AILD-Related Liver Necroinflammation

Notably, serum GP73 correlated with inflammatory activity grade (rho = 0.655, *P* < 0.001; rho = 0.547, *P* < 0.001), platelet count (rho = −0.422, *P* < 0.001; rho = −0.486, *P* < 0.001), and direct bilirubin (rho = 0.562, *P* < 0.001; rho = 0.480, *P* < 0.001) in patients with AIH or PBC. Consistent with the results of our previous study, GP73 correlated with the fibrosis stage in patients with AIH (rho = 0.713, *P* < 0.001) or with PBC (rho = 0.711, *P* < 0.001, Table 2). Moreover, multivariate analysis showed that after adjustment for serum PLT, ALT, AST, GGT, ALP, IgG, and IgM, serum GP73 remained an independent predictor of liver necroinflammation in both patients with AIH or PBC (Table 3).

### 3.4. Validation of the Diagnostic Value of Serum GP73 for AILD-Related Liver Necroinflammation

To further investigate whether GP73 would be clinically useful for the assessment of liver necroinflammation in patients with AILD, we calculated the AUROC, which indicated that serum GP73 was able to differentiate between the stages of fibrosis in patients with PBC or AIH. As shown in Table 4, the AUROCs for GP73 to predict moderate necroinflammation (≥G2) and severe necroinflammation (≥G3) in patients with AIH were 0.828 and 0.832, respectively, and the values for PBC were 0.797 and 0.802, respectively.

### 3.5. Comparison of the Diagnostic Values of Serum GP73 and ALP for the Assessment of Inflammation in Patients with PBC

**Figure 2:**
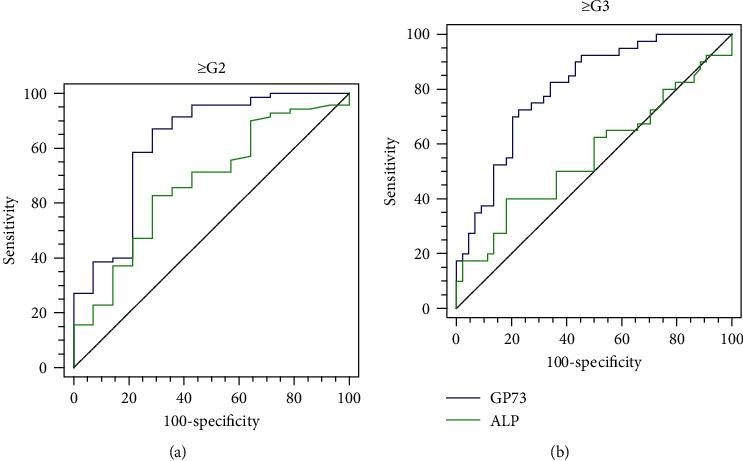
ROC curves of serum GP73 and ALP for moderate (a) and severe (b) necroinflammation in patients with PBC.

We compared the AUROC of serum GP73 with that of the widely used serum biomarker ALP for the assessment of the severity of necroinflammation in patients with PBC. As shown in Figure 2, the AUROCs of serum GP73 for the identification of moderate necroinflammation (≥G2) (AUROC = 0.820, *P* < 0.001) and severe necroinflammation (≥G3) (AUROC = 0.803, *P* < 0.001) were superior to those of ALP (≥G2: AUROC = 0.607, *P* = 0.028 and ≥G3: AUROC = 0.559, *P* = 0.357).

### 3.6. GP73 Was Upregulated by IL-6 in the Liver

**Figure 3:**
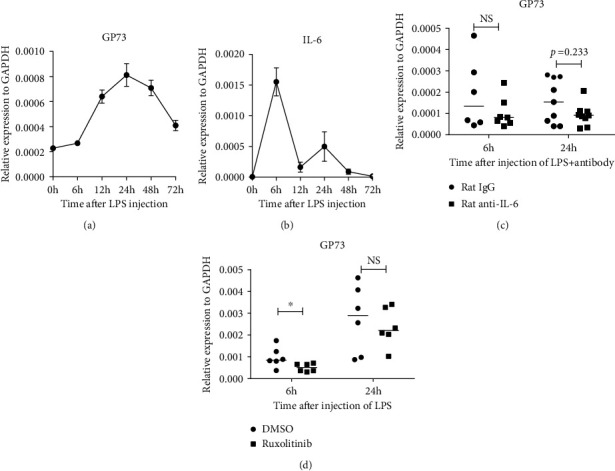
The mechanism for increased GP73 expression in liver inflammation. Quantitative real-time PCR detection of the mRNA levels of IL-6 (a) and GP73 (b) in C57/BL6J mice after LPS injection. Means ± SEM. *n* = 3. Quantitative real-time PCR detection of mRNA level of the GP73 in C57/BL6J mice after LPS injection or LPS plus IL-6 antibody (c) or ruxolitinib (d). Mean. *n* = 6. ^∗^*P* < 0.05, ^∗∗^*P* < 0.01, and ^∗∗∗^*P* < 0.001.

An LPS-induced liver injured C57/BL6J mouse model was established to imitate the occurrence of liver inflammation. After injection with LPS, hepatic mRNA levels of IL-6 were dramatically increased and peaked at 6 hours posttreatment (Figure 3(a)), which indicated that this acute hepatic inflammation model was successfully established. Meanwhile, GP73 increased in 12 hours (Figure 3(b)), just after the increase in IL-6. To further confirm the involvement of IL-6 (which has already been identified as the upstream regulator of elevated GP73 through ex vivo experiments in our previous study [23]), with rat IgG as a control, an IL-6 neutralizing anti-body was injected into C57/BL6 L mouse when LPS was injected. Hepatic elevated levels of serum GP73 post-LPS injection were inhibited in the presence of the IL-6 antibody, compared with the control group (Figure 3(c)). In our previous study [23], GP73 was upregulated by IL-6 through the STAT3 signaling pathway. To further confirm this hypothesis, ruxolitinib, which is an inhibitor of the JAK/STAT signaling pathway, was intragastrically injected into C57/BL6 L mice with simultaneous intraperitoneal injection of LPS. In agreement with our postulation, GP73 was decreased approximately 1-fold in the ruxolitinib treatment group compared to the control group (Figure 3(d)). These results suggested that the expression of GP73 was probably regulated by IL-6 when liver inflammation occurred.

## 4. Discussion

In the present study, we demonstrated that the level of serum GP73 increased with the severity of hepatic necroinflammation and is an independent risk factor for liver necroinflammation in patients with AILDs. Therefore, serum GP73 might represent an accurate biomarker of AILD-related liver necroinflammation, especially in AIH.

Because ALP is a widely accepted serum biomarker of liver inflammation in PBC, we compared the utility of serum GP73 for this purpose with that of ALP. AUROC analysis revealed that GP73 was superior to ALP for the prediction of moderate liver necroinflammation and disease severity. These results suggest that serum GP73 might represent a useful noninvasive biomarker for the assessment of prognosis in patients with AILD. Furthermore, because both AIH and PBC require long-term follow-up, serum GP73 might be useful for the long-term monitoring of patients and the efficacy of new drugs.

GP73 is normally expressed in biliary epithelial cells but rarely expressed in normal hepatocytes, and an increase in GP73 expression in hepatocytes has been reported by us and others in fibrotic/inflammatory damaged hepatic cells [16, 23, 24]. Since serum ALT elevation reflected the damage of hepatocytes, it is not difficult to understand the correlation of GP73 with ALT seen in AIH but not PBC patients (Table 2). Currently, the mechanisms underlying increases in GP73 are unknown. Previous studies have demonstrated that GP73 expression is affected by TGF-*β*, IL-1*β*, OSM, and IL-6 [5]. Of these, hepatic IL-1*β* and IL-6 expression was significantly higher in patients with AIH or PBC. IL-6 is a proinflammatory cytokine that is necessary for liver regeneration. The fact that GP73 is an IL-6 downstream factor may indicate that GP73 could play a role in IL-6-mediated liver inflammation and regeneration. In our previous study, we showed that the expression of GP73 is upregulated by IL-6 *via* the STAT3 signaling pathway [15]. LPS has been widely used to establish liver injury models [25]. In the present study, we used an LPS-induced mouse model to explore whether elevated GP73 in AILDs was dependent on IL-6. Our results in an LPS-induced mouse liver injury model demonstrated that hepatic GP73 was elevated just after the increase in IL-6, and blocking IL-6 by neutralizing the antibody or antagonizing the effects of IL-6 with the JAK/STAT signaling pathway inhibitor ruxolitinib partly inhibited the upregulation of serum GP73. This further confirmed that such proinflammatory cytokines may participate in the liver inflammation-related upregulation of GP73.

This study had several limitations. First, it was a retrospective analysis, and second, the sample size of 74 patients with AIH and 94 with PBC in the current study cohort was relatively small and might have led to sampling errors. A larger, prospective cohort study in the future would be warranted to confirm the present findings.

In conclusion, we have shown that serum GP73 is a simple and reliable biomarker of liver necroinflammation in AILDs.

## Figures and Tables

**Table 1:** Clinical characteristics of the patients.

Variable	AIH (*n* = 74)	PBC (*n* = 94)	*P* value
Male/female	13/61	15/79	0.781
Age (years)	49.5 ± 10.9	50.0 ± 10.3	0.829
Weight (kg)	60.0 ± 10.0	57.0 ± 8.2	0.013
PLT (10^9^/L)	181.8 ± 67.3	173.5 ± 83.0	0.478
ALT (UI/L)	198.1 ± 283.9	78.4 ± 90.5	<0.001
AST (UI/L)	151.8 ± 170.9	80.9 ± 83.2	0.002
ALP (UI/L)	153.8 ± 196.6	274.5 ± 242.3	0.001
GGT (UI/L)	152.1 ± 163.2	280.0 ± 373.2	0.005
TBIL (*μ*mol/L)	31.6 ± 49.2	30.6 ± 40.6	0.890
IgG (g/L)	16.5 ± 5.6	15.9 ± 5.9	0.523
IgM (g/L)	2.2 ± 2.6	4.2 ± 4.5	<0.001
GP73 (ng/L)	92.6 ± 59.7	83.5 ± 66.1	0.354
Fibrosis stage (0-1/2/3/4)	13/23/22/16	13/28/38/15	0.583
Liver necroinflammatory activity (0-1/2/3-4)	11/25/38	16/34/44	0.758

**Table 2:** Correlation between serum GP73 concentration and other parameters.

Variable	AIH (*n* = 74)		PBC (*n* = 94)	
	Rho	*P* value	Rho	*P* value
Male/female	0.213	0.069	-0.005	0.963
Age (years)	0.299	0.010	0.343	0.001
Weight (kg)	-0.027	0.832	0.079	0.469
PLT (10^9^/L)	-0.422	<0.001	-0.486	<0.001
ALT (UI/L)	0.311	0.007	0.034	0.748
AST (UI/L)	0.362	0.002	0.330	0.001
ALP (UI/L)	0.259	0.046	0.241	0.027
GGT (UI/L)	0.126	0.302	0.175	0.102
TBIL (*μ*mol/L)	0.416	<0.001	0.373	<0.001
DBIL (*μ*mol/L)	0.562	<0.001	0.480	<0.001
IgG (g/L)	0.336	0.003	0.223	0.039
IgM (g/L)	0.008	0.950	0.100	0.356
Fibrosis stage	0.713	<0.001	0.711	<0.001
Inflammatory activity grade	0.655	<0.001	0.547	<0.001

**Table 3:** Predictors of necroinflammation, according to multivariate analysis using a linear regression analysis model in patients with AIH or PBC.

Variables	Unstandardized coefficients		Standardized coefficients	*P* value
	*B*	Std. error	Beta	
AIH (constant)	1.811	0.183		<0.001
GP73	0.006	0.001	0.498	<0.001
GGT	0.001	0.001	0.284	0.022
PBC (constant)	1.921	0.284		<0.001
GP73	0.005	0.001	0.416	<0.001
IgM	0.033	0.012	0.297	0.008
PLT	-0.003	0.001	-0.314	0.009
ALP	0.001	<0.001	0.223	0.034

**Table 4:** Diagnostic value of GP73 for liver necroinflammation in AILD patients.

Stage	AUROC	Cut-off (ng/ml)	Sensitivity (%)	Specificity (%)	PPV (%)	NPV (%)	PLR	NLR
AIH (*n* = 74)								
≥G2 (63)	0.828 (0.723-0.906)	54.52	77.78	90.91	98.0	41.7	8.56	0.24
≥G3 (38)	0.832 (0.727-0.909)	65.68	81.58	77.78	79.5	80.0	3.67	0.24
PBC (*n* = 94)								
≥G2 (78)	0.797 (0.701-0.873)	48.19	74.36	75.00	93.5	37.5	2.97	0.34
≥G3 (44)	0.802 (0.707-0.877)	57.90	81.82	68.00	69.2	81.0	2.56	0.27

## Data Availability

Data are available on request.

## Abbreviations

AILD: Autoimmune liver disease
AIH: Autoimmune hepatitis
PBC: Primary biliary cholangitis
GP73: Golgi protein 73
ROC: Receiver operating characteristic
ALP: Alkaline phosphatase
HCC: Hepatocellular carcinoma
GGT: Gamma glutamyl-transpeptidase
ALT: Alanine aminotransferase
AST: Aspartate aminotransferase
TBIL: Total bilirubin
IgG: Immunoglobulin G
IgM: Immunoglobulin M
AUC: Area under the receiver operating characteristic curve
PPV: Positive predictive value
NPV: Negative predictive value
H&E: Hematoxylin and eosin
HRP: Horseradish peroxidase
IL-6: Interleukin-6.

## Supplementary Materials

Supplementary Materials Supplementary Table 1: Primers for real time quantitative PCR.
