# Supplementary material for: Diagnostic Value of Serum Golgi Protein 73 for Liver Inflammation in Patients with Autoimmune Hepatitis and Primary Biliary Cholangitis
Source: Dis Markers. 2022 Jan 15;2022:4253566. doi: 10.1155/2022/4253566 (PMC8783716; doi:10.1155/2022/4253566)
Supplement: Supplementary Materials — Supplementary Table 1: Primers for real time quantitative PCR. [file 4253566.f1.docx]

**Supplementary Table 1**

Supplementary Table1: Primers for real time quantitative PCR

| Gene name | Sequence (5`-3`) |
| --- | --- |
| m GP73 F | CCCAGTAGATGAATACGACATG |
| m GP73 R | GTCCATTTCACAGTTGAGTCAC |
| m IL-6 F | GCTTAATTACACATGTTCTCTGGGAAA |
| m IL-6 R | CAAGTGCATCATCGTTGTTCATAC |
| m GAPDH F | TGACCTCAACTACATGGTCTACA |
| m GAPDH R | CTTCCCATTCTCGGCCTTG |

*Abbreviation: m =mouse
